# Cost-effectiveness of Anti-VEGF treatments for age-related macular degeneration: a Brazilian perspective

**DOI:** 10.5935/0004-2749.20200020

**Published:** 2020

**Authors:** Renata Portella Nunes, Flávio Eduardo Hirai, Eduardo Buchelle Rodrigues, Michel Eid Farah

**Affiliations:** 1 Department of Ophthalmology and Visual Sciences, Escola Paulista de Medicina, Universidade Federal de São Paulo, São Paulo, SP, Brazil; 2 Instituto de Olhos de Florianópolis, Florianópolis, SC, Brazil

**Keywords:** Age-related macular degeneration, Cost-benefit analysis, Retina, Bevacizumab, Ranibizumab, Retina, Degeneração macular, Análise de custo-efetividade, Ranibizumabe, Bevacizumabe

## Abstract

**Purpose:**

To study the cost-effectiveness of ranibizumab and bevacizumab for the treatment of age-related macular degeneration.

**Methods:**

We used a decision tree model to analyze the cost-effectiveness of ranibizumab and bevacizumab for the treatment of age-related macular degeneration, from the Brazilian Public Health System (SUS) perspective. Ranib izumab and bevacizumab were administered to patients with the same treatment procedure, and the difference in treatment costs was calculated based on the cost of the drugs. Direct costs were estimated using the information provided by the Brazilian SUS. Effectiveness in terms of quality-adjusted life years (QALYs) was calculated based on the utility values for visual impairment. Incremental cost-effectiveness ratio was calculated by comparing both treatments. The analytical horizon was one year.

**Results:**

The decision tree analysis showed that the difference in treatment effectiveness was 0.01 QALY. Incremental cost-effectiveness ratio showed that ranibizumab treatment required an incremental annual cost of more than R$ 2 million to generate 1 additional QALY, as compared to bevacizumab.

**Conclusions:**

From the Brazilian SUS perspective, bevacizumab is more cost-effective than ranibizumab for the treatment of neovascular age-related macular degeneration. Its use could allow potential annual savings in health budget.

## Abstract (pt)

**Objetivo:**

Estudar o custo-efetividade do ranibizumabe e bevacizumabe no tratamento da degeneração macular relacionada à idade neovascular.

**Métodos:**

Utilizamos um modelo de árvore de decisão para analisar a relação custo-efetividade do ranibizumabe e bevacizumabe no tratamento da degeneração macular relacionada à idade, sob a perspectiva do Sistema Único de Saúde. O ranibizumabe e bevacizumabe foram administrados a pacientes com o mesmo procedimento de tratamento, e a diferença nos custos do tratamernto foi calculada com base no custo dos medicamentos. Os custos diretos foram estimados utilizando as informações fornecidas pelo SUS. A efetividade foi determinada em anos de vida ajustados pela qualidade (QALY) baseados em valores de utilidade em deficiência visual. A razão incremental custo-efetividade foi calculada comparando os dois tratamentos. O horizonte analítico foi de um ano.

**Resultados:**

A análise da árvore de decisão mostrou que a diferença na efetividade do tratamento foi de 0,01 QALY. A razão incremental de custo-efetividade mostrou que o tratamento com ranibizumabe exigiu um custo anual incremental de R$ 2 milhões para gerar um QALY adicional, em comparação ao bevacizumabe.

**Conclusões:**

Do ponto de vista do SUS, o bevacizumabe é mais custo-efetivo que o ranibizumabe no tratamento da degeneração macular relacionada à idade neovascular. O seu uso poderia gerar uma grande economia anual para o orçamento em saúde.

## INTRODUCTION

Age-related macular degeneration (AMD) is the major cause of irreversible visual impairment in elderly people worldwide and its treatment has become a great challenge for ophthalmologists^(1^,^2)^. The exudative AMD is characterized by an abnormal vascular ingrowth into the subretinal space and choroidal neovascularization, leading to sudden visual loss^(3)^.

Different treatments such as conventional laser photocoagulation and photodynamic therapy (PDT) with verteporfin and inhibitors of vascular endothelial growth factor (anti-VEGF agents) have been extensively studied in large prospective clinical trials. In recent years, anti-VEGF agents, which are able to improve visual acuity (VA), have emerged in the treatment of the exudative AMD. Among the anti-VEGF drugs, pegaptanib (Macugen; Eyetech/OSI, New York, NY), bevacizumab (Avastin^®^; Genentech, South San Francisco, CA), ranibizumab (Lucentis^®^; Genentech/Roche, South San Francisco, CA), and aflibercept (Eylea^®^; Regeneron, Tarrytown, NY) have been studied^(4^-^12)^.

Ranibizumab is a recombinant humanized monoclonal antibody fragment that binds and inhibits all biologically active forms of VEGF-A^(13)^. It was approved by the Food and Drug Administration for ophthalmological use in 2006. On the contrary, bevacizumab is a full-length recombinant humanized monoclonal antibody that binds and inhibits all biologically active forms of VEGF. It has not yet been approved for ophthalmological use. However, its comparable efficacy, safety, availability, and lower cost have promoted its off-label use as an alternative treatment to ranibizumab^(14)^.

Two pivotal studies comparing ranibizumab and bevacizumab were conducted in the United States and United Kingdom, which are known as the CATT study (*Comparison of Age-Related Macular Degeneration Treatments Trial*) and IVAN study (*Alternative treatments to Inhibit VEGF in Age-related choroidal Neovascularization*), respectively. After two years of investigation, both studies showed that ranibizumab and bevacizumab are equivalent in treatment efficacy and safety, if the same treatment strategy was used^(12^,^15^-^17)^. Other smaller clinical studies conducted in the United States, Austria, and Switzerland showed similar results^(18^,^19)^. Also, the first prospective comparative clinical trial of ranibizumab and bevacizumab for the treatment of AMD in the Brazilian population, which was reported by us, showed consistent results^(20)^.

Since exudative AMD causes central visual loss and metamorphopsia, it may significantly limit the abilities of patients to perform daily activities, such as reading and driving, thereby negatively affecting the patients’ quality of life. In addition, treatment with costly drugs increases healthcare costs, thus creating social and economic hardships in the healthcare system.

As previously mentioned, some studies have already demonstrated that ranibizumab and bevacizumab are comparable in terms of safety and efficacy^(12^,^15^-^17)^. The greater efficacy and cost-effectiveness of anti-VEGF treat ments, as compared to other therapies such as PDT and pegaptanib injections, have been well-demonstrated in the literature^(21)^. The IVAN study concluded that ranibizumab is not cost-effective as compared to bevacizumab. Ranibizumab showed a very high cost without a significant gain in the quality adjusted life years (QALYs)^(22)^. Other studies in the United States also demonstrated the high cost-effectiveness of bevacizumab as compared to ranibizumab^(23)^.

A budget impact analysis of the Brazilian Public Health System (SUS) has recently been performed. It was based on a systematic review of the literature about the treatment options (PDT, ranibizumab, and bevacizumab) and a meta-analysis of the prevalence of AMD in the Brazilian population, which estimated 284,000 cases of exudative AMD between 2008 and 2011. Due to the savings generated, the introduction of bevacizumab was recommended for the treatment of exudative AMD in the Brazilian SUS^(24)^.

A cost-effectiveness analysis directly comparing ranibizumab and be vacizumab has not yet been conducted within the Brazilian population. In health economics, the perspective of this study is important because of the costs and benefits involved in the analyses. There has been an increasing demand from the Brazilian government to understand better the economic impact of incorporating new health technologies and treatments in the Brazilian SUS. Thus, the present study was conducted to analyze the cost-effectiveness of ranibizumab and bevacizumab for the exudative AMD treatments from the Brazilian SUS perspective.

## METHODS

In the present study, we compared monthly injections of ranibizumab with monthly injections of non-repacked bevacizumab. A decision tree model was used as the basis of economic analysis using TreeAge Pro^®^ software (TreeAge Software Inc, Williamworth, MA, USA). From each treatment option, each model had three outcomes based on VA: improvement, stability, or decrease (Fi gure 1). VA improvement was determined when the patient gained 15 or more letters in the ETDRS VA chart^(25)^; stability was defined as a change, positive or negative, of no more than 14 letters; and decrease was defined as a loss of 15 letters or more. All transition probabilities used in the decision tree were based on the CATT study results^(12)^ (Table 1).

**Table 1:** Transition probabilities used in the decision tree models comparing monthly injections of ranibizumab and bevacizumab

	VA improvement	VA stability	VA decrease
Ranibizumab	0.34	0.60	0.06
Bevacizumab	0.31	0.63	0.06

VA= visual acuity.

Based in data from CATT study.^(12^,^15)^

### Analytical horizon

Analytical horizon is a determined time period during which the economic analysis is performed. In this analysis, an analytical horizon of one year was used because there were no studies showing if there was VA improvement or decrease in periods longer than two years. A simulation of a longer time period was done in our sensitivity analysis.

### Effectiveness

For the drug effectiveness, we calculated QALY, which is a disease burden measurement, where it incorporates not only the quality (morbidity) but also the quantity of life years (mortality). It is a universal form to valuate an individual’s life and disease, allowing comparisons between different health conditions.

In this study, to calculate QALY, we used utility measurements. All utility values were based on the previous studies on visual impairment^(26)^ (Table 2).

**Table 2:** Utility values and QALYs for each VA outcome

	Utility	Interval
VA improvement	0.81	0.73-0.89
VA stability	0.57	0.47-0.66
VA decrease	0.52	0.38-0.66

VA= visual acuity; QALY= quality-adjusted life years.

Adapted from Patel JJ et al.^.^ and Brown et al.^(26^,^27)^

### Costs

The cost analysis was performed using the Brazilian SUS perspective. Direct costs related to the AMD treatment were the costs of medical appointments, the drugs ranibizumab and bevacizumab, and supplies (syringe, needle, eye drops). Appointment costs were obtained from the SUS costs chart and the costs of medications and supplies were obtained from the Ministry of Health database^(28)^. Ranibizumab and bevacizumab costs were obtained from the list of drugs for public purchase of the Agência Nacional de Vigilância Sanitária (ANVISA). This list shows a great variety of prices due to the tax on movement of goods and services between different Brazilian states. Therefore, we decided to use the cost without considering taxation. The unitary cost of each injection for each treatment was calculated. Indirect costs such as the ones related to individual productivity loss and intangible costs were excluded from this analysis. We followed the monthly injection protocol used by the CATT study for both drugs^(15)^. All the analyses were presented in Brazilian Reais (R$).

**Figure 1:**
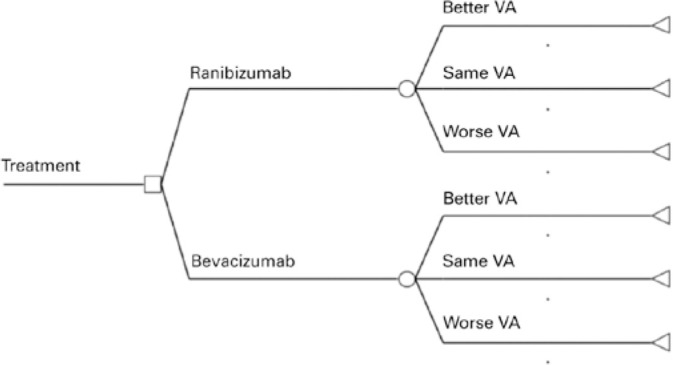
Decision tree model used in this cost-effectiveness study comparing ranibizumab and bevacizumab.

VA= Visual acuity

The incremental cost-effectiveness ratio (ICER) was calculated, which represents the cost per QALY gained by the patient by comparing the two drugs using the formula:

### Sensitivity analysis

In an economic evaluation, a sensitivity analysis takes into account the change in a variety of parameters in the economic model and observing their impact on the results. In this study, we chose to evaluate the following parameters in the analysis:

Transition probabilities and every biweekly bevacizumab injection strategy, according to the data from a clinical trial recently performed by our department at the Federal University of São Paulo (UNIFESP)^(20)^;

Other forms of drug application according to the CATT study: monthly ranibizumab, monthly bevacizumab, as-needed ranibizumab, and as-needed bevacizumab. As-needed injections were based on the clinical parameters during the follow-up of the study participants, and the ophthalmologist decided if the medication should be applied or not. In this sensitivity analysis, we evaluated the strategies that implied changes in the number of injections per year in each patient’s treatment;

Five-year analytical horizon. The clinical trial conducted in our department has shown that the mean age of the study patients was approximately 75 years. Accordingly, we decided to use an analytical horizon of five years, which we believe is an adequate number, considering the life expectancy of patients with AMD in Brazil. We used an annual discount rate of 5% following the recommendations of the methodological guidelines for economic evaluation of health technologies by the Health Ministry^(29)^. This discount rate considers the influence of time on costs and various consequences. We assumed that all patients with AMD should follow the drug application protocol throughout this period and that the VA would be stable after the first 12 months of treatment.

Variation in the number of injections that could be applied with one vial of bevacizumab, in the case of repacking.

## RESULTS

Table 3 shows the unitary and annual costs relative to AMD treatment. The only cost difference between the two drugs was the drug value. The total unitary cost per injection of ranibizumab was R$ 2,206 and that of bevacizumab was R$ 950.

**Table 3:** Monthly costs related to the AMD treatment using monthly ranibizumab and bevacizumab injections

	Unitary cost (R$)	Annual cost (R$)
Direct costs	15.23	182.76
Bevacizumab	934.84	11,218.08
Ranibizumab	2,190.85	26,290.20

Unitary cost= vial cost for 1 injection; Annual cost= cost calculated for 12 injections.

Table 4 shows the cost-effectiveness evaluation of monthly ranibizumab and bevacizumab injections. The total annual cost (vial cost + direct costs) of the ranibizumab treatment was R$ 26,472 and Bevacizumab treatment was R$ 11,401. The CATT study showed that there was a no significant difference in effectiveness between the two drugs^(12)^.The decision tree analysis showed that the difference in effectiveness was 0.01 QALY, 0.65 and 0.64 for ranibizumab and bevacizumab, respectively. The incremental cost for ranibizumab injection was R$ 15,072. ICER showed that ranibizumab treatment requires an incremental annual cost of more than R$ 2 million to generate 1 additional QALY, as compared to bevacizumab. Despite the cost differences, none of the treatment strategies was better.

**Table 4:** Cost-effectiveness evaluation of the ranibizumab and bevacizumab monthly injections

Treatment	Cost in 1 year (R$)	QALYs	Incremental cost (R$)	ICER (R$/QALY)
Ranibizumab	26,472.46	0.65	15,071.62	2,093,350.00
Bevacizumab	11,400.84	0.64	0.00	

QALY= quality adjusted life years; ICER= incremental cost-effectiveness ratio.

### Sensitivity analysis

Analysis 1: Applying the results from the UNIFESP clinical trial^(20)^.

To evaluate the impact on the results, we also decided to use the transition probabilities obtained in the study performed at UNIFESP in our analysis (Table 5).

**Table 5:** Transition probabilities used in the decision tree models comparing ranibizumab and bevacizumab injections in the UNIFESP comparative clinical trial

Treatment	VA improvement	Stability	VA Worsening
Monthly bevacizumab	0.20	0.73	0.07
Biweekly bevacizumab	0.13	0.80	0.07
Monthly ranibizumab	0.20	0.80	0.00

VA= visual acuity.

Another strategy used in the UNIFESP study was the biweekly bevacizumab injections. This strategy had no significant difference in efficacy and safety when comparing monthly treatment. However, there was a non-significant greater tendency towards resolution of PED with biweekly treatment^(20)^. The incremental cost for ranibizumab injections was R$ 3,671. ICER showed that monthly ranibizumab treatment warrants an incremental annual cost of R$ 180,851 to generate 1 additional QALY, as compared to biweekly bevacizumab treatment. Thus, even with a greater number of injections and biweekly follow-up (with as-needed injections), the bevacizumab strategy continued to be more cost-effective.

Analysis 2: CATT study treatment strategies

We evaluated the cost-effectiveness of three other treatment strategies used in the CATT study. For each case, the transition probabilities were adjusted according to the study results.

1. Monthly ranibizumab versus as-needed bevacizumab: 12 ranibizumab injections/year and 8 bevacizumab injections/year.

In this case, despite the higher cost of ranibizumab strategy than that of bevacizumab, due to the higher number of injections, ranibizumab showed a slightly greater effectiveness, 0.65 versus 0.63 QALY. The bevacizumab strategy continued to be more cost-effective. 2. As-needed ranibizumab versus monthly bevacizumab: 7 ranibizumab injections/year and 12 bevacizumab injections/year

In this strategy, even with the greater number of bevacizumab injections, there was a higher cost for ranibizumab and there was no dominance of either of the strategies

3. As-needed ranibizumab versus as-needed bevacizumab When the as-needed strategy was used, where patients received drug injections according to clinical evaluation, neither drug presented dominance.

Analysis 3: Five-year analytical horizon

Using a 5-year analytical horizon and discount rate of 5%, ICER showed that to have 1 additional QALY, when comparing ranibizumab versus bevacizumab, an additional cost of more than R$ 2 million per year would be necessary. Neither strategy has shown dominance. We also performed a sensitivity test changing the discount rate from 0 to 10%, and we came to the same conclusion.

Analysis 4: Variation in the number of injections with one bevacizumab vial.

It is well-known that a bevacizumab vial may be used for more than one injection, allowing it to be used for more than one patients. Thus, we performed an analysis varying the cost of bevacizumab according to the number of patients treated with one vial. The first simulation was performed using a vial for 10 injections and then for 20, 30, and 40 injections. For this calculation, we added the repackaging cost to the direct costs and the cost of the drug fraction:

### Unitary cost = Repackaging cost + Direct costs + Vial/number of fractions

Table 6 shows the total bevacizumab unitary cost (per injection) according to the number of injections per vial.

**Table 6:** Total bevacizumab unitary cost (per injection) after considering repackaging

Number of fractions		Unitary cost (R$)
10		128.81
20		81.97
30		66.40
40		58.60

## DISCUSSION

Since the first reported use of bevacizumab in 2005, the off-label use of bevacizumab for AMD treatment has increased worldwide because of its low cost. Its use has increased after the evidence of the non-inferiority of this drug in comparison with ranibizumab in the CATT and IVAN studies^(12^,^15^-^17)^.

As the commercially available vial is superior to the necessary intravitreal dose, the repackaging of bevacizumab becomes possible and attractive when considering the cost reduction. However, repackaging could increase the risk of contamination, besides a hypothetical reduction in the efficacy of the drug.

This economic evaluation indicated that bevacizumab is more cost-effective than ranibizumab. Other published studies found similar results^(23)^. Raftery et al. evaluated the cost-effectiveness of the two drugs from a British health system (NHS - National Health System) pers pective, using cost data from 2005^(30)^. Their results showed that ranibizumab was not cost-effective when compared to bevacizumab. Ranibizumab would have to be 2.5 times more effective to be more cost-effective than bevacizumab. They also demonstrated that the adverse events had a minimal impact on the cost-effectiveness values. The limitation of this study was the lack of comparative data between the two drugs at that time.

Another cost-effectiveness study comparing the two drugs was conducted by Patel et al. from an American health system perspective^(27)^. These authors demonstrated that bevacizumab use was 95% more cost-effective than ranibizumab in neovascular AMD treatment. In this study, the cost-effectiveness ratio was USD 1,405 for QALY of bevacizumab and USD 12,177 for QALY of ranibizumab. The incremental cost between the two drugs was USD 55,649. In other words, it would be necessary to spend more than USD 55,000 per year to get one additional QALY per patient, if ranibizumab was used as treatment.

There has been an analysis of the budgetary impact of neovascular AMD treatment options (PDT, ranibizumab, and bevacizumab) from a SUS perspective. Introduction of bevacizumab was recommended due to the cost savings achieved^(24)^.

The present study analysis showed an incremental cost-effectiveness ratio of more than R$ 2 million in ranibizumab treatment. The comparison of these results with the previous studies is not possible nor recommended, considering that they were performed with different populations. Also, the contextual differences in drug effectiveness and costs definitions limit the comparisons.

The dominance of one drug over another occurs if one of them is less effective and has a higher cost. The lower cost strategy predominates over the higher cost when there is equivalence in effectiveness. There was no dominance of any strategy evaluated in this study. The effectiveness values included were based on the CATT study. This study showed no statistically significant differences in effectiveness of the strategies used^(12)^. Therefore, bevacizumab may be considered as more cost-effective since its cost is lower. Considering the much lower cost and repackaging, we concluded that the best strategy for neovascular AMD treatment was the bevacizumab treatment.

In this analysis, we did not consider the possible complications of either treatment, such as postoperative infection or intraocular hemorrhage, among others. It is known that complications may modify the treatment course, increasing costs and consequently influencing the economic evaluations. However, the procedures related to the analyzed treatments are identical, and the only difference is the type of drug being injected. Studies have demonstrated that complication rates are very similar for the two drugs^(7^,^10^,^12)^. Furthermore, complication rates were very low which would not have a significant impact on outcomes^(7^,^10^,^12)^.

A major limitation of our study is that the measurements of quality of life and utility were extrapolated from the American studies, as this type of information among Brazilians was not available. Despite the limitations, this is the first Brazilian cost-effectiveness comparison between ranibizumab and bevacizumab from a SUS perspective.

This economic evaluation indicated that bevacizumab is more cost-effective than ranibizumab in the treatment of neovascular AMD, from a SUS perspective. On the basis of the data presented, the introduction of bevacizumab in the treatment of neovascular AMD could be recommended due to greater cost-effectiveness and annual savings potential in health budget.
